# Amelioration of non-motor dysfunctions after transplantation of human dopamine neurons in a model of Parkinson's disease

**DOI:** 10.1016/j.expneurol.2016.02.003

**Published:** 2016-04

**Authors:** M.J. Lelos, R.J. Morgan, C.M. Kelly, E.M. Torres, A.E. Rosser, S.B. Dunnett

**Affiliations:** Brain Repair Group, School of Biosciences, Cardiff University, Cardiff, Wales CF10 3AX, UK

**Keywords:** Parkinson's disease, Non-motor symptoms, Visuo–spatial tasks, Cell transplantation, Ventral mesencephalon, Dopamine

## Abstract

**Background:**

Patients suffering from Parkinson's disease (PD) display cognitive and neuropsychiatric dysfunctions, especially with disease progression. Although these impairments have been reported to impact more heavily upon a patient's quality of life than any motor dysfunctions, there are currently no interventions capable of adequately targeting these non-motor deficits.

**Objectives:**

Utilizing a rodent model of PD, we investigated whether cell replacement therapy, using intrastriatal transplants of human-derived ventral mesencephalic (hVM) grafts, could alleviate cognitive and neuropsychiatric, as well as motor, dysfunctions.

**Methods:**

Rats with unilateral 6-hydroxydopamine lesions to the medial forebrain bundle were tested on a complex operant task that dissociates motivational, visuospatial and motor impairments sensitive to the loss of dopamine. A subset of lesioned rats received intrastriatal hVM grafts of ~ 9 weeks gestation. Post-graft, rats underwent repeated drug-induced rotation tests and were tested on two versions of the complex operant task, before post-mortem analysis of the hVM tissue grafts.

**Results:**

Post-graft behavioural testing revealed that hVM grafts improved non-motor aspects of task performance, specifically visuospatial function and motivational processing, as well as alleviating motor dysfunctions.

**Conclusions:**

We report the first evidence of human VM cell grafts alleviating both non-motor and motor dysfunctions in an animal model of PD. This intervention, therefore, is the first to improve cognitive and neuropsychiatric symptoms long-term in a model of PD.

## Highlights

• Non-motor dysfunctions affect quality of life in Parkinson's disease.
• We tested whether human-derived foetal dopamine cells could improve these deficits.
• Human dopamine cells improved rotational bias and movement impairments in a rat model.
• Non-motor dysfunctions, specifically visuospatial and motivational deficits, improved.
• This is the first evidence of improved non-motor deficits from human dopamine cells.

## 1 Introduction

In Parkinson's disease (PD), a common neurodegenerative disorder characterized neuropathologically by Lewy body formation and deterioration of the nigrostriatal pathway, patients present with motor, cognitive and neuropsychiatric symptoms. The non-motor dysfunctions are frequently reported as reducing quality of life and impacting on the well-being and health status of people with PD to a greater degree than any motor complications (Duncan et al., 2014, Hinnell et al., 2012). Non-motor symptoms are increasingly recognized as core dysfunctions of the disease, and indeed are typically observed prior to the onset of overt motor impairments (Hawkes, 2008, Meissner, 2012). Multiple aspects of function can become compromised, including impaired executive function, increased apathy, depression, impaired visuo–spatial abilities, and reduced motivation.

The effective alleviation of non-motor dysfunction is a critical element of any effective therapy, but there are currently no interventions that are capable of adequately targeting both motor and non-motor impairments. While pharmacological therapies, such as l-dopa, alleviate some motor dysfunctions in the majority of patients, they often have no impact upon, or can even impair, cognitive performance (Hernández et al., 2014, Obeso et al., 2011, Schneider et al., 2013, Zibetti et al., 2013). Systemic administration of dopamine-enhancing drugs is unlikely to improve cognitive dysfunctions effectively, given that both too little and too much dopamine are known to impair neural processing, and that optimal levels differ across different sub-regions of the brain (Cools and D'Esposito, 2011).

Cell replacement therapies hold considerable promise as a therapeutic intervention. In open-label clinical studies, intrastriatal grafts of foetal ventral mesencephalon (hVM; the region of the human foetal brain in which the nigrostriatal dopamine cells develop) produced long-term improvements in motor function, removing the need for pharmacological interventions in some patients (Freed et al., 1992, Kefalopoulou et al., 2014, López-Lozano et al., 1997, Wenning et al., 1997, Lindvall, 1997). Transplants of hVM have provided proof-of-principle that implantation of dopamine neurons can produce functional benefit and are pivotal in paving the way for transplantation of stem cell-derived dopamine neurons. However, little is known about the ability of hVM grafts to alleviate any non-motor dysfunctions observed in PD patients. Cognitive disability has not been comprehensively assessed in many clinical trials of transplantation in PD, making it difficult to determine the impact of cell therapies on this functional output (Ostrosky-Solís et al., 1988, Sass K et al., 1995).

Results obtained from rodent PD models, utilizing rat-derived VM, have revealed some improvements in visuo–spatial performance (Dowd and Dunnett, 2004, Heuer et al., 2013). While these studies provide encouraging data regarding the ability of cell therapies to target one aspect of non-motor impairment, it has yet to be revealed whether hVM can endow such effects (Lelos et al., 2012) and whether multiple domains of non-motor dysfunctions can be improved. Although several studies report long-term survival of hVM tissue in the rodent brain (Brundin et al., 1986, Strömberg et al., 1989, Strömberg et al., 2001, Strömberg et al., 2010), few studies investigated the functional efficacy of these grafts. For example, it has been reported that hVM grafts of 6–9 weeks gestation can reduce drug-induced rotational bias (Brundin et al., 1986, Brundin et al., 1988, Clarke et al., 1999, Grealish et al., 2010, Strömberg et al., 1995, Strömberg et al., 1986). What has not been demonstrated is the capacity of these cells to impact upon the more debilitating non-motor dysfunctions that manifest in PD. Thus, the aim of this study was to investigate the functional impact of hVM grafts on non-motor and motor impairments.

## 2 Methods

To test our hypothesis, rats were pre-trained on a complex operant choice reaction time task in a 9-hole box apparatus, which measures visuospatial function and incentive motivation. Rats received lesions of the nigrostriatal pathway, by infusion of the selective neurotoxin 6-hydroxydopamine. After the grafting of hVM ectopically into the dorsal striatum, rats were tested for drug-induced rotation, followed by testing on two versions of the choice-reaction time task.

### 2.1 Subjects

Twenty-six female Lister-hooded rats (Charles River, UK) were maintained on a 14:10-h light/dark cycle. All rats were food restricted one week before commencing operant training/testing and for the duration of any operant experimental work, in order to motivate learning in the operant task. During food restriction, rats were maintained at 90% of their baseline weights. All experiments were conducted in compliance with the UK Animals (Scientific Procedures) Act 1986 under Home Office Licence No. 30 ⁄ 2498 and with the approval of the local Cardiff University Ethics Review Committee.

### 2.2 Apparatus

#### 2.2.1 Operant chambers

The 9-hole chambers (Paul Fray, U.K.) were constructed of aluminium (25 × 25 cm) with a grid floor, and the back wall housed an array of nine holes, each of which contained a light-emitting diode to provide a visual stimulus, and a vertical infrared beam with a photocell detector, which detected nose poke responses into the hole. A food magazine in the middle of the opposite wall signalled the delivery of 45-mg sucrose reward pellets (TestDiet, IN, USA). On-line data collection was controlled by BNC software (Campden Instruments, UK).

#### 2.2.2 Rotometers

A bank of 16 automated rotometer bowls (Rotorat, Med Associates, VT) (Ungerstedt and Arbuthnott, 1970) recorded the frequency of amphetamine-induced rotation for 90 min after i.p. injection of 2.5 mg/kg methamphetamine hydrochloride (Sigma-Aldrich, UK). Apomorphine-induced rotations were recorded for 60 min after subcutaneous injection of 0.05 mg/kg apomorphine hydrochloride hemihydrate (Sigma-Aldrich, UK). Rotation scores expressed as an average of ipsilateral minus contralateral rotations.

### 2.3 Experimental design

Rats (n = 26) were pre-trained for 7 weeks in the 9-hole operant chambers on the Bilateral choice reaction time task. A cohort of rats (n = 18) received infusion of 6-OHDA into the MFB. Eight rats remained as unoperated controls. At 2 weeks post-lesion, all rats underwent amphetamine-induced rotations and were tested in the operant box apparatus for 5 days to measure lesion-induced deficits. Rats were separated into equal groups (lesion only versus grafted) based on rotation and operant performance. One cohort of lesion rats (n = 10) then received intrastriatal transplantations of hVM tissue. All rats were immunosuppressed daily with intraperitoneal injections of 10 mg/kg cyclosporine (Sandoz Pharmaceutical, U.K.). Amphetamine-induced rotations were tested at 3 week intervals. At 14 weeks post-graft, rats were re-tested in the operant boxes, using two distinct versions of the task. At 20 weeks, rats underwent an apomorphine-induced rotational challenge and were then perfused and brains tissue taken for histological analysis.

### 2.4 Behavioural testing

**Fig. 1:**
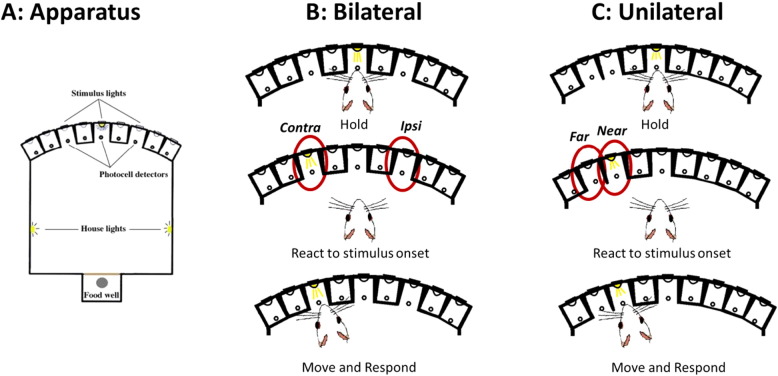
Schematic representation of the two versions of the operant choice reaction time task. The Bilateral version (left) required the rat to hold its nose in the centre hole of the array until a stimulus light flashed briefly on the ipsilateral or contralateral side. The Unilateral version (right) was similar, except that the stimuli were both situated on the side of the body contralateral to the lesion, with one hole in the same location as the Bilateral version (Near), and the other one hole further away (Far).

Pre-training was conducted as previously described (Lindgren et al., 2014) (Fig. 1). Briefly, the central location in the array and a lateral “response” location one space away on the left and right of the central hole were utilized. Rats were trained to hold their noses in the central location for a variable delay to initiate brief presentation (200 ms) of a lateralised light. A correct response into the lateralised hole triggered delivery of a sucrose pellet into the magazine (45 mg, TestDiet, IN, USA). Errors were recorded and resulted in a 2 s time out. Failure to maintain hold in the central location for the variable delay duration resulted in a time out and an unusable trial. The session duration was 30 min.

The behavioural outputs were calculated as follows. *Trials usable*: trials in which the rat responded to the illuminated centre hole for the required delay, initiating the presentation of the stimulus light. *Accuracy*: percentage of correct responses, divided by the total number of responses made into the two available locations. *Reaction time*: mean latency to initiate a response by withdrawal of the nose from the centre hole location after the onset of the lateral stimulus light (on correct responses only). *Movement time*: mean latency to execute the lateralised nose poke response after removal of the nose from the centre hole (on correct responses only).

Post-graft, rats were tested in alternating one week blocks on the version of task described above (‘Bilateral’) and on an alternative version of the task (‘Unilateral’). The Unilateral version of the task, in which both response locations are located on one side only, was used to probe response choice within contralateral space (Heuer et al., 2013, Ungerstedt and Arbuthnott, 1970). This more challenging version probes visuo–spatial performance by revealing more precisely the ability of the rat to generate and execute responses on the side of the body contralateral to the dopamine-depleting lesion. The same response location on the left was utilized (now referred to as ‘Near’) and the space immediately to the left of it was exposed (‘Far’).

### 2.5 Surgical procedures

#### 2.5.1 Lesions

Rats were anaesthetised with isoflourane (2–4% with carrier gases oxygen and nitrous oxide) in a stereotaxic frame. The MFB was targeted with an injection of 12 μg (freebase) of 6-OHDA (Sigma; 3 μl of 4 μg/μl solution at 1 μl/min with 2 min diffusion) in 0.01% ascorbate saline at stereotaxic coordinates AP − 4.4 and ML ± 1.0 (from bregma) and DV − 7.8 below dura.

#### 2.5.2 Transplantation

Human foetal tissue was collected from medical terminations of pregnancy with full donor consent, through the SWIFT human foetal tissue bank (http://www.biobankswales.org.uk/swift-research-tissue-bank), under UK Human Tissue Authority research licence (no. 12457) held by Cardiff University, and with ethical approval of the project from the Bro Taf local research ethics committee. Gestational age was estimated through ultrasound scan prior to the procedure in combination with measurement of foetal regions (Evtouchenko, 1996).

The hVM tissue was harvested from four foetuses of ~ 9 weeks gestation, with a mean (and standard deviation) crown-rump length of 26.4 mm (± 1.96). Tissue was used as individual suspensions and not pooled. Tissue was incubated at 37 °C for 20 min in TryplE Express (Life Technologies Inc) containing 20 U/ml Dornase alfa (Pulmozyme, Roche). Tissue was dissociated by trituration in 200 μl of DMEM/D to obtain a quasi-single cell suspension. The viability for each sample was estimated as > 95%. Each rat received 2 × 2 μl suspension containing approximately 500,000 cells in total. Cells were implanted at a rate of 0.5 μl/min and distributed over two stereotaxic sites (AP: + 0.5, ML: − 3.0 mm and AP: + 1.2, ML: − 2.6 mm) at depths of − 4.5 and − 3.7 mm.

### 2.6 Perfusion

Rats were terminally anaesthetized with sodium pentobarbital (Euthatal, Merial, UK) and sacrificed by transcardial perfusion with 0.01 M phosphate buffer followed by 1.5% paraformaldehyde (pH 7.4; Sigma-Aldrich). Brains were post-fixed for 24 h in 1.5% PFA before being transferred to 25% sucrose solution. Tissue was sectioned on a freezing sledge microtome at 40 μm thickness in a 1:12 series.

### 2.7 Bright-field and fluorescent immunohistochemistry

Free-floating sections were blocked in 3% normal serum, then incubated in primary antibody with 1% serum overnight at RT. Tissue was incubated in secondary antibody (1:200) with 1% serum for 3 h, then immersed in an avidin-biotinylated enzyme complex (Vector Laboratories) for 2 h, then either stained with DAB or Vector SG (Vector Laboratories).

The primary antibodies used in either bright-field or fluorescent immunohistochemistry were tyrosine hydroxylase (TH, 1:1000, Millipore, AB1542); Girk2 (1:500, Alomone, APC-006); Calbindin (1:1000, Sigma, C9848), and HuNu (1:1000, Millipore, MaB 1281).

### 2.8 Statistics

Statistical analyses were conducted using SPSS (v20, IBM) or, to adjust for missing values, GenStat (v16, VSN International). Newman Keuls method was used for post-hoc analyses. One grafted rat was removed from the behavioural data due to not responding in the operant box during the post-graft testing period.

## 3 Results

### 3.1 Histological analyses

**Fig. 2:**
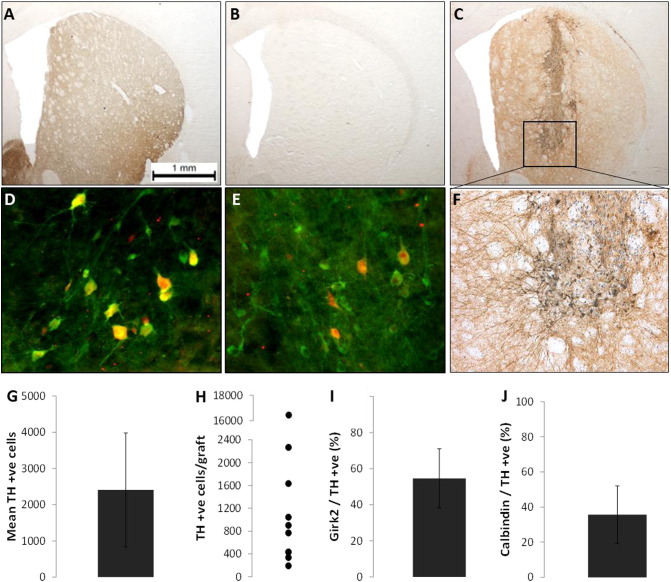
Immunohistological analysis of hVM tissue at 20 weeks post-graft. Immunohistochemistry of TH + ve neurons (brown) and HuNu (blue) in the hVM graft (top panel). From left to right, images depict representative tissue from a Control rat (A), Lesion rat (B) and a large hVM graft (C). The central panel depicts A9 TH + ve neurons (green) co-labelled with Girk2 (red, D); A10 TH + ve neurons (green) co-labelled with Calbindin (red, E); × 10 magnification of hVM cells with TH + ve neurons stained in brown and HuNu + ve cells in blue (F). The bottom panel depicts the number of TH + ve cells per individual graft (G) and as a group mean (H), as well as the proportion of girk2 + ve (I) and calbindin + ve (J) cells out of the total number of TH + ve neurons. Scale bar = 1000 μm. Error bars = ± standard error of mean. (For interpretation of the references to colour in this figure legend, the reader is referred to the web version of this article.)

At 20 weeks post-graft, surviving dopamine-rich grafts were revealed by staining for tyrosine hydroxylase (TH; see Fig. 2). On average, there were over 2000 TH positive cells in each graft, with over 50% of these labelling as Girk2 positive (a marker for A9 nigral subtype of dopamine neurons) and ~ 40% were Calbindin + ve (a marker for A10 ventral tegmental subtype of dopamine neurons). The variability in the number of cells surviving is likely related to variability between donors. TH + ve fibres were observed innervating the host tissue, with a pronounced bias towards projecting to the medial region of the striatum. Some innervation of TH positive fibres into the ventral striatum was also evident.

### 3.2 Drug-induced rotational bias

**Fig. 3:**
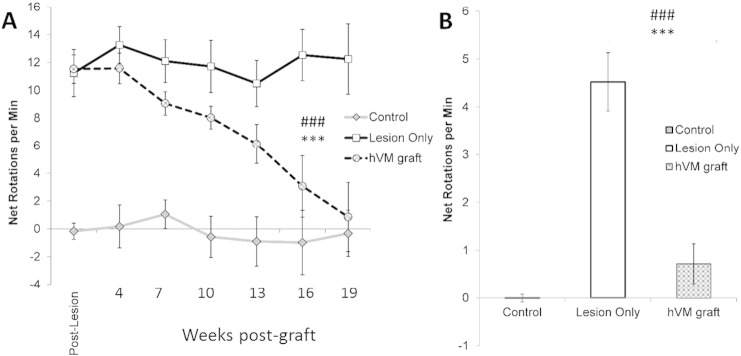
hVM grafts reduce drug-induced rotational bias. Mean net rotations per minute for rats injected i.p. with 2.5 mg/kg methamphetamine (A). Rotational bias was measured post-lesion, then at 3 weekly intervals, between 4 and 19 weeks post-graft. Apomorphine was injected subcutaneously at 0.05 mg/kg in a single rotational test, which was conducted at 20 weeks post-graft (B). Significant effect of Group: ***p < 0.001. Significant difference between hVM grafted rats and Lesion rats: ^###^p < 0.001. Error bars = ± standard error of mean.

In the amphetamine-induced rotation task (Fig. 3A), Lesion rats maintained a consistent rotational bias across the test sessions. However, hVM grafted rats demonstrated a progressive reduction in rotation, until performance was identical to Control rats [Group ∗ Week: F_12,138_ = 4.71, p < 0.001; effect of Week for hVM rats, p < 0.001]. In the apomorphine rotation test (Fig. 3B), hVM grafted rats rotated significantly less than Lesion rats, and instead performed comparably to Control rats [Group: F_2,23_ = 33.36, p < 0.001; Lesion vs Control and hVM: all p < 0.001; Control vs hVM, n.s.].

### 3.3 Bilateral choice reaction time task

**Fig. 4:**
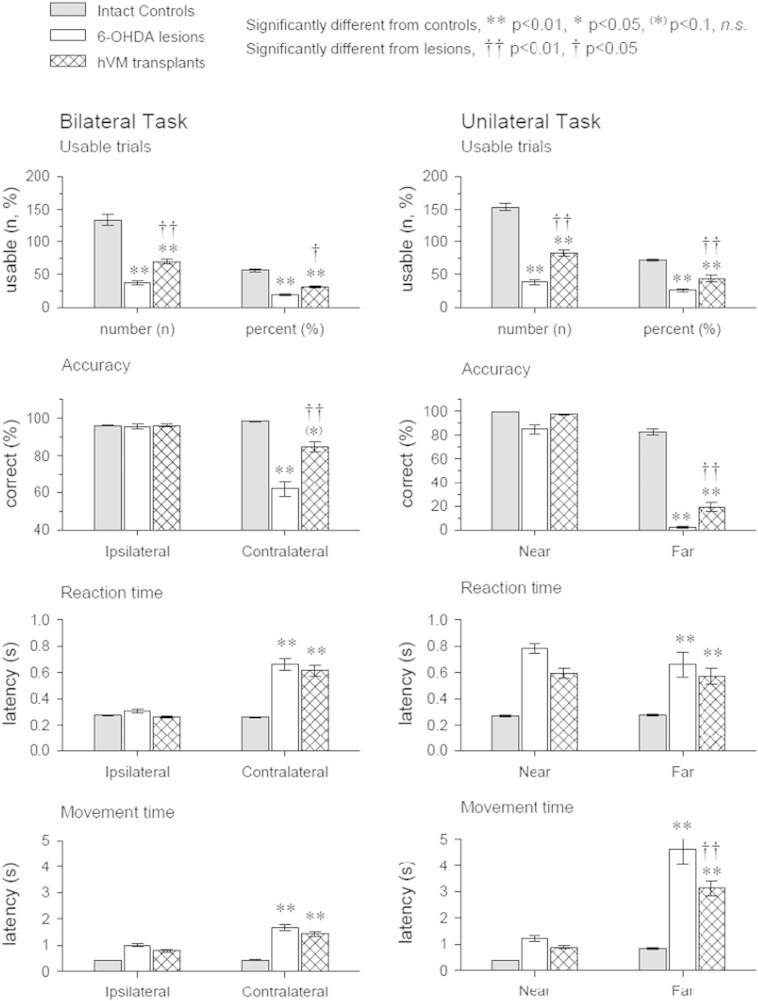
hVM grafts improve non-motor and motor performance on an operant task. Performance during the final week (18 weeks post-graft) on the Bilateral version (left column) and the Unilateral version (right column) of the choice reaction time task. Data portray the total number of usable trials and the percentage of total trials that were usable, accuracy, latency to react and latency to respond. Significant difference from Control rats: **p < 0.01, *p < 0.05, (*)p < 0.1 (n.s.). Significant difference from Lesion rats: ^††^p < 0.01, ^†^p < 0.05. Error bars = ± standard error of mean.

Data from the last week of the Bilateral version of the task, 18 weeks post-graft, are presented in Fig. 4. The results reveal improved execution of usable trials and improved accuracy in rats grafted with hVM tissue, relative to lesion rats.

#### 3.3.1 Usable trials

Rats grafted with hVM tissue made significantly more usable trials than the Lesion cohort, and a greater percentage of their total trials were usable, revealing the ability of hVM tissue to improve the rats' motivation to initiate trials [Fig. 4A; Group: F_2,22_ = 40.0 and 46.82, respectively, ps < 0.001; hVM vs Lesion, ps < 0.05].

#### 3.3.2 Accuracy

On the contralateral side, hVM grafts significantly improved accuracy relative to the Lesion rats, making the performance comparable to Control rats [Fig. 4B; Group ∗ Side: F_2,16_ = 9.38, p < 0.005; on contra side, Lesion vs Control and hVM, ps < 0.05; hVM vs Control, p < 0.05].

#### 3.3.3 Reaction time

On the contralateral side, the reaction time was significantly slower for both Lesion and hVM rats, compared to control [Fig. 4C; Group ∗ Side: F_2,16_ = 8.29, p < 0.001].

#### 3.3.4 Movement time

The latency to move to the response location was longer on the contralateral side for Lesion and hVM grafted rats, compared to control [Fig. 4D; Group ∗ Side: F_2,16_ = 9.51, p < 0.005].

### 3.4 Unilateral choice reaction time task

Data from the last week of the Unilateral version of the task, 19 weeks post-graft, are presented in Fig. 4. The results reveal improved execution of usable trials, improved accuracy and faster response times in rats grafted with hVM tissue, relative to lesion rats.

#### 3.4.1 Usable trials

hVM rats successfully completed significantly more usable trials than the Lesion cohort and a greater percentage of total trials were usable [Fig. 4E; Group: F_2,22_ = 84.01, p < 0.001, hVM vs Lesion ps < 0.01].

#### 3.4.2 Accuracy

Rats grafted with hVM tissue demonstrated significantly greater response accuracy than Lesion rats [Fig. 4F; Group ∗ Side: F_2,16_ = 28.08, p < 0.001, hVM vs Lesion hVM, p < 0.01].

#### 3.4.3 Reaction time

The latency to react was shorter for Control rats than Lesion or hVM rats [Fig. 4G; Group: F_2,22_ = 12.72, p < 0.001]. No effect of hVM graft was evident on the reaction time.

#### 3.4.4 Movement time

On the far hole, hVM graft rats were significantly faster to respond than Lesion rats [Fig. 4H; Group ∗ Side: F_2,16_ = 18.92, p < 0.001, Lesion vs hVM, p < 0.01].

## 4 Discussion

In this study, we tested the hypothesis that human-derived cell replacement therapies can alleviate non-motor cognitive and neuropsychiatric deficits that manifest in neurodegenerative disease states. We believe this to be the first clear evidence that hVM transplants have the capacity to improve performance on non-motor aspects of impairment associated with the loss of dopamine innervation, in a rat model of PD.

The histological findings, including number of surviving human-derived TH + ve neurons, the proportion of A9/A10 subtypes, and the degree of histological variability, was in line with previous observations (Grealish et al., 2010). Significant innervation of the dopaminergic projections into the host tissue was observed, with a bias towards innervation of the more medial neostriatum and ventral striatum. Two distinct tests of drug-induced rotation revealed significant alleviation of rotational bias in rats grafted with hVM tissue, indicating that hVM grafts survived, integrated into tissue and released sufficient dopamine to improve the severe turning bias induced by unilateral MFB loss. However, because these tests bear little relevance to the clinical situation, it remained important to evaluate the functional efficacy of the hVM tissue on tasks which necessitated skilled motor function and which comprised elements of non-motor function.

The choice reaction time task (Dowd and Dunnett, 2004, Heuer et al., 2013) isolates the involvement and processing capacity of a single hemisphere during a trial. This way, we can observe a within-subject deficit unilaterally in the rat and performance on motor and non-motor elements of the task can clearly be dissociated. Although the Bilateral version of the task reveals impairment in responding to the contralateral side induced by loss of dopamine, the Unilateral version of the task demonstrates that, under alternative conditions, lesion rats are capable of generating motor responses in contralateral space.

The latency to move to, and respond in (movement time), the far hole was shorter in hVM grafted rats than in lesioned animals in the Unilateral version of the task. A trend towards reduced movement time can be observed numerically in the Bilateral version, although this effect did not reach significance. Taken together with the results of the drug-induced rotational tests, this suggests that overt motor dysfunctions were broadly improved by the presence of the hVM graft. The latency to react (reaction time) was not alleviated by the presence of the hVM grafts, which is in accordance with previous studies that utilized rat-derived VM grafts (Dowd and Dunnett, 2004, Heuer et al., 2013). This suggests that attentional deficits have not been improved by the hVM transplantation.

More interestingly, we observed an improvement in several of the non-motor aspects of function in the hVM grafted rats. It has been demonstrated that initiation and completion of usable trials is dependent upon the propensity to initiate goal-directed behaviours and on the motivational status of the rat (Lelos et al., 2012). Thus, reducing incentive motivation by specific-satiety of the sucrose reward has been shown to reduce the production of goal-directed behaviour and, consequently, significantly decrease the number of trials executed (Lelos et al., 2012). Loss of nigrostriatal dopamine does not disrupt reward or hedonistic processing per se, but rather it decreases the motivation to work for a reward (Drui et al., 2014, Favier et al., 2014, Schmidt et al., 2008, Shiner et al., 2012). In accordance with this, PD patients present with deficits in mood, decreased motivation and apathetic behaviour (Aarsland et al., 2005). The data presented here demonstrates that reinnervation of dopaminergic projections into the striatum from hVM grafts resulted in increased numbers of trials being initiated and completed, which reveals enhanced motivational status and improved goal-directed performance.

The hVM grafted rats also demonstrated improved accuracy of performance relative to Lesion rats. It has been suggested that loss of dopamine impairs the ability to direct responses in contralateral space, potentially as a result of processing a distorted representation of response space (Brown and Robbins, 1989, Brown and Robbins, 1991). Our data demonstrate that while contralateral responses are impaired in Lesion rats in the Bilateral task, they are able to respond with high accuracy in the same response hole (previously impaired location; near hole) when a second, more distal, response location is exposed (Unilateral version). Thus, responding into the near hole is not disrupted per se, but instead the ability to direct actions in space is impaired. It is possible to conclude, therefore, that the deficit in responding to the far, but not the near, hole is a result of an inability to code movements appropriately in space, while the ability to generate movement remains intact (Brown and Robbins, 1989, Brown and Robbins, 1991).

The hVM grafts alleviated both the motor and non-motor deficits. Although similar findings have been observed after transplantation of rat-derived VM, this is the first evidence of hVM grafts alleviating these deficits. Indeed, while rat VM improved non-motor impairments at 12 weeks post-graft (Dowd and Dunnett, 2004, Heuer et al., 2013), we now find that hVM requires a longer (18–20 weeks) maturation period before functional recovery is observed in these tasks of non-motor function.

The recovery of these behaviours may be explained by the specific pattern of innervation and the heterogeneous nature of the striatum. Medial regions of the striatum support cognitive and goal-directed behaviours, as would be expected from the pattern of innervation from the association cortex areas. Conversely, the lateral striatum is important for motor function and receives heavy afferent connections from motor cortex regions. Additionally, the observed innervation of the ventral striatum may account for the improvement in non-motor dysfunctions. The mesolimbic dopamine pathway, which comprises efferent dopaminergic VTA projections to the ventral striatum (nucleus accumbens), is a critical system involved in motivation and incentive salience (Haber, 2014). Indeed, to further support this, Piccini and colleagues found that PD patients that showed the least clinical improvement demonstrated early or continued loss of ventral striatal dopamine, relative to patients that benefited most who had more intact mesolimbic pathways (Piccini et al., 2005). Histological observation of the tissue grafts revealed greater propensity for the dopaminergic projections to innervate the medial and ventral striatum. While the reason for this innervation pattern remains unknown, we can nevertheless deduce that the improvements in non-motor function may be driven by this pattern of subregional recovery.

The operant task probes non-motor functions that have been shown to be disrupted in PD patients, where changes in mood, decreased motivation and apathy are commonly seen with disease progression (Aarsland et al., 2005). It has been suggested that the loss of motivational processing that underlies decreased goal-directed behaviour in rodents is akin to apathetic behaviour in patients (Levy and Dubois, 2006). Apathy is known to impact severely on an individual's ability to function normally, and thus improvement in this neuropsychiatric symptom is critical for patients with PD. Here we have shown for the first time that alleviation of apathetic-like behaviour, and the consequent reinstatement of voluntary goal-directed behaviours, can be observed after implantation of hVM grafts.

Visuospatial function is disrupted in PD, insofar as the ability to appropriately direct responses in space is reduced. Patients demonstrate visuospatial deficits shown to be related to higher-order perceptual motor dysfunction, rather than disruption of fine motor control (Richards et al., 1993). As a result, it has been suggested that patients inappropriately utilize sensory information accurately to plan and execute complex movements (Richards et al., 1993). Critically, we have demonstrated the functional efficacy of hVM grafts by revealing an improvement in visuospatial processing capabilities in rats that received the cell therapy.

It is increasingly recognized that patients with PD not only suffer from a range of non-motor dysfunctions, but also that these often manifest before the onset of motor symptoms and can be some of the most debilitating aspects of the disease (Hawkes, 2008, Meissner, 2012). Pharmacological interventions are typically not capable of alleviating these non-motor dysfunctions. Dopaminergic drugs improve motor dysfunctions when first administered to patients, but the effect on neuropsychological function is less clear. Improvements in a set-switching task have been reported after administration of levodopa (Cools et al., 2001, Cools et al., 2003), but also significantly impaired probabilistic reversal learning (Cools et al., 2001) and impaired decision making due to increased impulsivity (Cools et al., 2003). On two reaction time tasks, apomorphine was reported to impair reaction time performance (Müller et al., 2002, Costa et al., 2003). Given that each brain region has different basal and optimal levels of dopamine, it is unsurprising that systemic administration would cause imbalance in other regions, resulting in diverse changes in neuropsychiatric and executive functions (Cools and D'Esposito, 2011).

After chronic ingestion of these drugs, initial improvements were reported on several tasks, which ultimately worsened over time (Kulisevsky et al., 2000). Other studies report no improvement in cognitive function after long-term levodopa, or levodopa plus dopamine agonist, therapy (Rektorová et al., 2005, Relja and Klepac, 2006). Interestingly, however, it has recently been shown that intrajejunal infusion of levodopa over 6 months may improve some non-motor symptoms, including sleep, attention/memory and gastrointestinal issues (Honig et al., 2009). Although improvements in perception, mood and cognition did not meet conventional levels of significance in this study, a trend was observed (Honig et al., 2009). Thus, there is little evidence to suggest that oral administration of dopaminergic drugs, as acute or chronic treatments, may be a suitable therapy for alleviating the non-motor dysfunctions that manifest in PD.

Cell replacement therapies have an advantage insofar as replacement of lost neurons can be targeted to each patient according to specific patterns of denervation, thereby allowing more precise reinnervation of the neurotransmitter. Using this method, we have demonstrated the ability of hVM grafts to alleviate cognitive, neuropsychiatric and motor impairments. This opens up the real possibility of utilizing human-derived cell therapies to target the non-motor impairments that manifest in PD. Thus, by improving non-motor deficits, it may be possible to improve the quality of life for people with PD.

## Author contributions

MJL, AER, and SBD designed the experiment. MJL, RM, CMK, and EMT ran the experiment and analysed the data. MJL, RM, CMK, EMT, SBD, and AER wrote the manuscript.

## Potential conflicts of interest

Mr Morgan, Drs Lelos, Kelly and Torres, and Professors Rosser and Dunnett reported no biomedical financial interests or potential conflicts of interest.
